# Efficacy and Damage Diagnosis of Reinforced Concrete Columns and Joints Strengthened with FRP Ropes Using Piezoelectric Transducers

**DOI:** 10.3390/s22218294

**Published:** 2022-10-29

**Authors:** Chris G. Karayannis, Emmanouil Golias, Maria C. Naoum, Constantin E. Chalioris

**Affiliations:** Laboratory of Reinforced Concrete and Seismic Design of Structures, Civil Engineering Department, School of Engineering, Democritus University of Thrace, 67100 Xanthi, Greece

**Keywords:** piezoelectric lead zirconate titanate (PZT) transducers, string linear variable displacement transducers (SLVDTs), reinforced concrete (RC), structural health monitoring (SHM), beam-column joint (BCJ), carbon fiber-reinforced polymer (C-FRP) ropes, real-scale tests

## Abstract

Recent research has indicated that the implantation of a network of piezoelectric transducer patches in element regions of potential damage development, such as the beam–column joint (BCJ) area, substantially increases the efficacy and accuracy of the structural health monitoring (SHM) methods to identify damage level, providing a reliable diagnosis. The use of piezoelectric lead zirconate titanate (PZT) transducers for the examination of the efficiency of an innovative strengthening technique of reinforced concrete (RC) columns and BCJs is presented and commented on. Two real-scale RC BCJ subassemblages were constructed for this investigation. The columns and the joint panel of the second subassemblage were externally strengthened with carbon fiber-reinforced polymer (C-FRP) ropes. To examine the efficiency of this strengthening technique we used the following transducers: (a) PZT sensors on the ropes and the concrete; (b) tSring linear variable displacement transducers (SLVDTs), diagonally installed on the BCJ, to measure the shear deformations of the BCJ panel; (c) Strain gauges on the internal steel bars. From the experimental results, it became apparent that the PZT transducers successfully diagnosed the loading step at which the primary damage occurred in the first BCJ subassemblage and the damage state of the strengthened BCJ during the loading procedure. Further, data acquired from the diagonal SLVDTs and the strain gauges provided insight into the damage state of the two tested specimens at each step of the loading procedure and confirmed the diagnosis provided by the PZT transducers. Furthermore, data acquired by the PZT transducers, SLVDTs and strain gauges proved the effectiveness of the applied strengthening technique with C-FRP ropes externally mounted on the column and the conjunction area of the examined BCJ subassemblages.

## 1. Introduction

Damaging seismic excitations worldwide have clearly pointed out the inadequate behavior of reinforced concrete (RC) buildings designed and constructed according to older codes and practices. Insufficient steel reinforcement, or even total lack of steel stirrups in critical regions of structural elements, have often resulted in extended damage and total failures [1].

The structural parts that, in the majority of the observed cases, have long been identified as critical elements for damage initiation, and partial, or even total, seismic failure, are the beam-column joints (BCJ) [2]. It has been emphasized that, as a result of the poor detailing of BCJs, their brittle response is frequently observed after severe earthquakes and there have been many cases where this behavior has led to partial or total collapse of a structure [3]. Evidently, research on the development and establishment of a strengthening technique, suitable for deficient RC elements, is a scientific field of high priority and significance, due to the necessity for buildings to be safe and the relevant important impact if they are not.

The older, and most frequently applied, strengthening method is concrete jacketing with conventional steel reinforcing bars [4]. In recent decades fiber-reinforced concrete and fiber-reinforced polymer (FRP) composite materials have been used for the retrofitting of deficient RC structural members subjected to seismic or cyclic loading [5,6,7,8,9,10]. Each technique includes special detailing and different labor levels and disruption periods in the operation of the structure. In general, the use of FRP sheets minimizes the application problems encountered during the construction of the RC jacketing. Nevertheless, anchoring of the FRP sheets has been proven to be a problem for the efficiency of this strengthening technique. Cases of U-form jacketing have also been experimentally investigated and have proved that the anchoring of the FRP sheets is a critical parameter [11,12].

Reviews presenting the state-of-the-art of BCJs strengthened with FRP sheets and tested in cyclic loading have been reported in the literature [13]. Furthermore, experimental works, investigating near-surface-mounted FRPs and new steel products, such as near surface mounted (NSM) methods, have also been repeatedly reported [14]. Dalfre and Barros [15] reported successful NSM techniques for the strengthening of slabs, too. Other forms of materials and techniques, like fully-fastened-haunches [16], steel meshes [17], external bars [18], and steel plates with shear connectors [19], have also been experimentally investigated for the upgrading of RC elements and BCJs. Analytical efforts have also been reported, as well [20,21].

Furthermore, the use of FRP reinforcing bars with carbon fibers have been investigated as an alternative to traditional steel bars [22,23]. Important advantages of this novel material, in comparison to the steel bars, are its higher tensile strength, its light weight and its highly corrosive-resistant nature. Quite recently, carbon FRP, in the form of flexible ropes, were experimentally investigated as externally applied strengthening reinforcement of RC BCJs, by Murad et al. [24], and the authors in [25,26,27]. The use of this innovative material as additional strengthening reinforcement for the upgrading of RC BCJs, deficient or not deficient, is a promising new approach, due to its advantages, since it is a rapid and easily applied retrofitting method.

The use of FRP ropes and their application as external reinforcement for the strengthening of both columns and the BCJ of RC frames is experimentally investigated herein. Moreover, for in-depth examination of the efficacy of the proposed strengthening scheme, piezoelectric lead zirconate titanate (PZT) transducers are employed. It is emphasized that the usage of PZTs not only gives valuable information about the response of the strengthened subassemblage and the efficiency of the strengthening technique, but can also be used for structural health monitoring of the applied strengthening scheme and the subassemblage as a whole.

Within the recently developing technology of structural health monitoring (SHM) of RC structures, papers have been devoted to the field of damage identification, localization and assessment [28,29,30,31]. Acoustic emission testing is a well-known, and widely used, non-destructive method that monitors, detects and locates imperfections and damage in mechanically loaded materials and structural members. However, global vibration-based approaches are insensitive to local pristine structural damage, due to the nature of lower-order modal parameters. Some local inspection methods, such as acoustic emission and X-rays, have been examined for the health investigation of RC members. Strain-based methods, via fiber optical sensor-embedded smart strands, are a practical way of detecting the variation of tensile forces in RC members.

Among various local SHM techniques, impedance-based methods have been used to estimate the development of concrete compressive strength and for structural damage detection due to concrete cracking, steel reinforcement yielding and/or force quantification. In these techniques, electromechanical impedance signals are gained from PZT transducers via coupling interactions of the transducers and the host structure. Since impedance signatures characterize the structural features of the diagnosed region, the change in structural properties leads to variation in impedance responses. Any local deterioration could be detected by computing variations in impedance signals measured before and after a damaging event [32].

Further, compared with contact-based non-destructive methods, the implementation of PZT sensors, ultrasonic sensors, electromagnetic acoustic transducers and accelerometers with noncontact testing modalities have proved to be more promising for in-situ wave-based damage detection. In recent years, ultrasonic waves excited by PZT sensors and/or electromagnetic acoustic sensors have been applied to monitor defects due to debonding, delamination and corrosion between steel bars and concrete. Damage localization can be achieved using time reversal and multi-path probing methods. However, antisymmetric and axisymmetric modes have been observed in cases where the material properties are identical and symmetrical. The characteristic of the guided wave depends on the geometrical shape. For these reasons, more research is required to improve damage diagnosis in terms of accurately and efficiency based on the appropriate excitation signals [33].

Advanced SHM techniques implementing advanced transducers and smart materials have been proven to be capable of providing continuous structural damage detection in RC elements [34,35]. PZT patches have been widely applied in the recently proposed SHM approaches because of their advanced and very useful characteristics, such as small size and ease of application, low cost, and their dual functional features, acting as actuators and sensors simultaneously with quick response [36,37,38,39]. 

Recent research has indicated that the implantation of a network of PZT patches in element regions of potential damage development substantially increases the efficacy and the precision of the SHM methods to diagnose level of damage level and provide a reliable diagnosis [40,41,42,43,44]. In particular, Perera et al. [45] and Sevillano et al. [46] extended PZT-based SHM by developing an advanced clustering approach that satisfactorily evaluates several measurements from the PZT transducers network and diagnoses damage in RC elements strengthened with FRP materials. In beams strengthened with FRP sheets, Liu et al. studied debonding failure based on PZT patches [47].

In this work two real-scale BCJ specimens were constructed for the needs of this investigation. The columns and the joint panel of the second BCJ subassemblage were externally strengthened with FRP ropes. In the first subassemblage, three PZTs were epoxy-bonded on the concrete surface in the diagonal direction of the joint body. In the second BCJ subassemblage three PZT patches were also epoxy-bonded. Two of them were bonded on the diagonal C-FRP ropes that strengthened the joint body and one on the concrete surface.

In this study, first, the effectiveness of using small-sized PZT transducers for diagnosing the damage of BCJ specimens subjected to cyclic loading was examined. The magnitude of the differences in the voltage values at each step of the loading procedure was representative of the changes brought about in the material in the neighborhood of the PZT during the loading step, indicating the level of damage that occurred. Damage detection using PZT-based SHM techniques in RC structural members that suffered seismic or quasi-static loading has not been thoroughly examined so far, although earthquake damage in critical RC members, such as BCJ, reduce the capacity of structures to withstand future seismic excitations and increase the risk of catastrophic collapses if not controlled.

Further, we investigated the efficiency of the retrofitting technique using different types of transducers: (a) String linear variable displacement transducers (SLVDTs) diagonally mounted on the BCJ for the measurement of the shear deformations of the joint panel; (b) Strain gauges on internal steel reinforcing bars; (c) PZT sensors on the composite ropes and the concrete.

To the author’s best knowledge, the use of PZT sensors in C-FRP ropes for continuous monitoring and the prompt diagnosis of fatal debonding of the composite reinforcement, or shear diagonal cracking of joint concrete, has not been previously examined. Further, for the first time, the efficiency of this strengthening technique was assessed by applying traditional displacement transducers and strain gauges, along with smart materials such as PZT sensors.

Furthermore, this study aimws to provide solid indications that the SHM technique can offer real-time, continuous inspection and in-situ measurement for far-field damage identification and early warning of imminent failure in real-life RC frame buildings strengthened with C-FRP ropes.

## 2. Design of the BCJ Specimens

### 2.1. Characteristics of the BCJs

**Figure 1:**
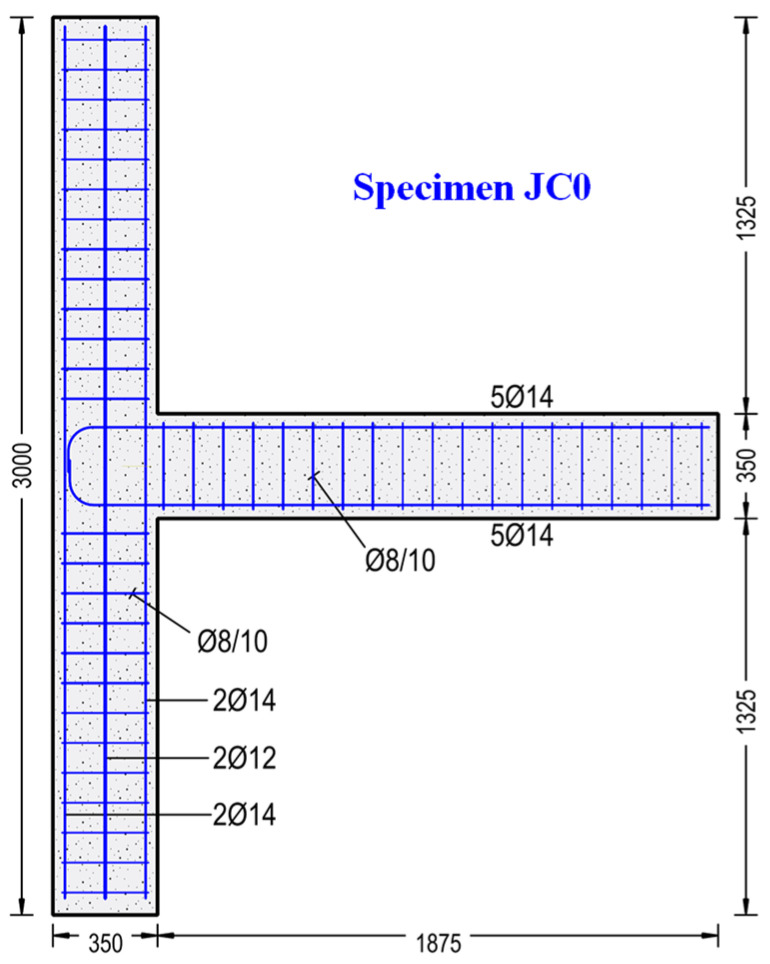
Specimen JC0. Geometrical and reinforcement characteristics.

Geometry, materials and reinforcements of the tested subassemblages were selected and designed to be representative of common frame RC structures. The length of the column was equal to 3.0 m, with cross-sectional dimensions of 350/250 mm. The length of the beam was 1.875 m, with cross-sectional dimensions of 350/250 mm. Geometrical and reinforcement characteristics of specimen JC0 are presented in Figure 1.

**Figure 2:**
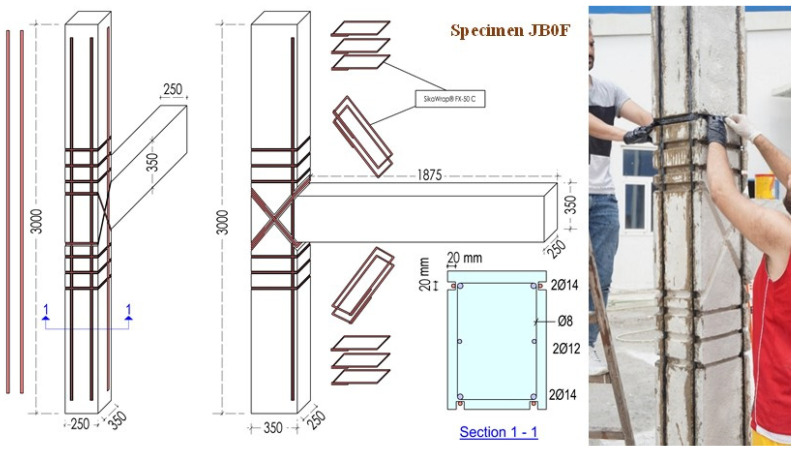
Dimensions and strengthening scheme applied on specimen FB0F. Ropes were added at the four corners of the column along the height, as external longitudinal reinforcement, and, further, ropes were added round the column as external confining stirrups at the critical zones of the column and, finally, diagonally placed C-FRP ropes were mounted on each one of the two sides of the joint as strengthening reinforcement of the joint body.

The aim of the test project was to investigate the effectiveness of the application of C-FRP ropes as additional strengthening, when diagonally placed on each one of the two sides of the BCJ subassemblages, in the following: (i) longitudinal and transverse reinforcement of the column and (ii) shear reinforcement of the joint body. In this direction, specimen JB0F was retrofitted with C-FRP ropes added as external NSM in the following ways: (a) Longitudinal tensional reinforcement along the height of the column; (b) Confining transverse shear reinforcement around the column in the critical regions near the joint; (c) Diagonal shear reinforcement of the joint at its two sides. The strengthening scheme is presented in Figure 2.

The compressive strength of concrete was evaluated by supplementary compression tests of six standard cylinders (150 × 300 mm) as *f_cm_* = 34 MPa. The mean tensile strength of the conventional steel reinforcement was *f_y_* = 550 MPa. The C-FRP rope was a bundle of unidirectional carbon fibers with a tensile strength equal to 4000 MPa, modulus of elasticity equal to 240 GPa and cross-sectional area > 28 mm^2^, according to the manufacturer’s data (SikaWrap^®^ FX-50C and SikaWrap^®^ FX Fibre Connector, SIKA HELLAS SA, Kryoneri, Attica, Greece) [48,49]. Epoxy resin type A (Sikadur^®^-52, SIKA HELLAS SA, Kryoneri, Attica, Greece) was used for the impregnation of the C-FRP rope dry fibers and epoxy resin type B (Sika AnchorFix^®^-3+, SIKA HELLAS SA, Kryoneri, Attica, Greece) was used to anchor the composite ropes.

### 2.2. External Installation of the Strengthening C-FRP Ropes

The applied retrofitting technique included processes that have to be meticulously performed [50]. Initially, lines of the exact location of the C-FRP ropes had to be drawn on the concrete surface in order to direct the cutting and incision of the U-shaped notches with dimensions of depth/width approximately equal to 25/30 mm. Meticulous cleaning of the notches, using compressed air, was also required. External corners of the notch lines had to be rounded to a radius of at least 20 mm, based on the manufacturer’s recommendations [48,49]. The existing conventional steel reinforcing bars and stirrups had to be protected from unintentional damage during notch formation and further attention was essential to ensure and secure each C-FRP rope anchorage [51,52].

### 2.3. Examination of the Expected Damage

The failure mode of the BCJ subassemblages was studied, based on the well-established model developed by Tsonos [53]. According to this model, the ultimate shear stress *τ_ult_* of the joint and the factor $\gamma_{ult}=\tau_{ult}/\sqrt{f_{cm}}$ are first determined and, then, compared to the developing shear stress *τ_cal_* and the corresponding factor $\gamma_{cal}=\tau_{cal}/\sqrt{f_{cm}}$. From this comparison, it was deduced that the expected damage and critical cracking would be localized in the joint body, a fact that was verified during the cyclic loading tests.

## 3. Test Setup and Instrumentation

**Figure 3:**
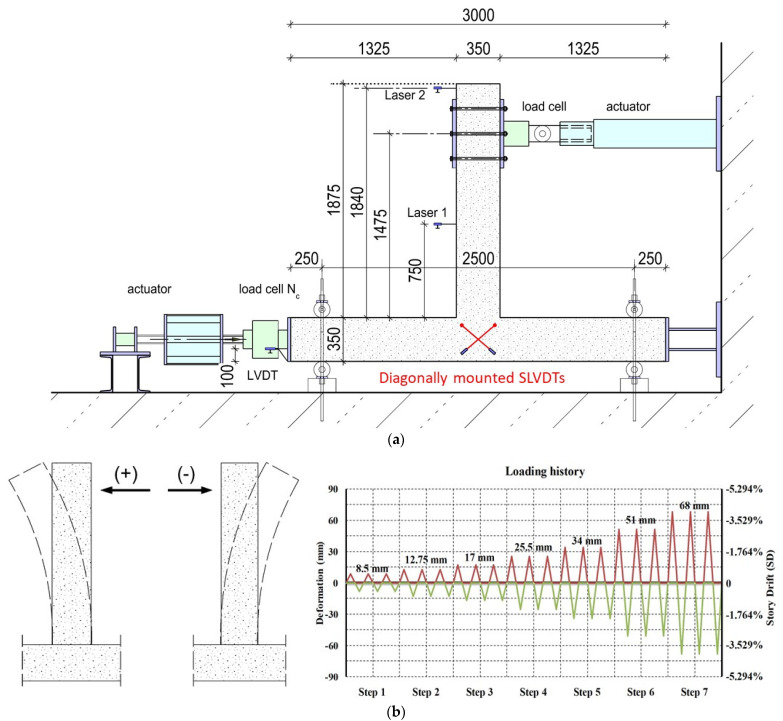
Experimental setup and loading history: (**a**) The column of the BCJ specimen was placed in the horizontal direction and the beam in the vertical direction. The deformation was horizontally imposed near the free end of the beam (dimensions in mm); (**b**) The load sequence included 7 steps and each step comprised three equal full loading cycles.

The test setup and the imposed cyclic loading sequence are shown in Figure 3a,b, respectively. The tested specimens were rotated 90 degrees, so that during the testing procedure the column was in the horizontal direction and the beam in the vertical direction (Figure 3a). During the experimental procedure, a constant axial compressive load, equal to *N_c_* = 0.05, *A_c_ f_cm_*, was applied in the column of the subassemblage. Both specimens were subjected to the same cyclic in-plane displacement control loading in a strong and rigid RC floor–wall testing area. The column of the RC BCJ specimens was adequately fixed to the floor with bolts and nuts to prevent the specimen slipping.

The load was imposed at the end of the beam of the specimens using a double-acting servo-controlled hydraulic actuator fixed to the rigid wall of the laboratory with a maximum capacity of 500 kN and 500 mm total stroke, connected to an intermediate hydraulic control unit to ensure its smooth operation.

Deformations of the tested BCJ specimens were presented in terms of story drift (SD) that represented the ratio of the imposed deformation to the length from the loaded end of the beam to the column centerline. All specimens were subjected to the same loading history of full cyclic deformations.

The cyclic loading sequence included seven steps with imposed deformations: ±8.50 mm, ±12.75 mm, ±17.00 mm, ±25.50 mm, ±34.00 mm, ±51.00 mm and ±68.00 mm. The imposed deformations corresponded to the following respective SDs: 0.50%, 0.75%, 1.00%, 1.50%, 2.00%, 3.00% and 4.00%. Each loading step comprised three full loading cycles, as illustrated in Figure 3b.

The response characteristics of the tested subassemblages were measured using the following measuring instruments: (a) Load shell for the measurement of the imposed displacement and loading near the free end of the beam (Figure 3a); hysteretic responses were drawn and presented in this study; (b) SLVDTs diagonally mounted on the BCJ for the measurement of the shear deformations of the joint panel; (c) Strain gauges applied on internal steel bars recorded the strains of steel bars during the loading; (d) PZT transducers applied on the ropes and the concrete for the damage diagnosis of ropes and the concrete.

## 4. Diagonal SLVDTs and Measurement of Shear Deformations

During seismic excitations, shear deformations develop in the joint bodies of BCJs of multi-story RC frame structures. The shear deformations of the joints constitute a practical indication of cracking, and, thereby, the damage level. Further, shear deformations can be considered a valuable way to assess the effectiveness of the examined retrofitted technique to improve the seismic performance of the BCJ.

**Figure 4:**
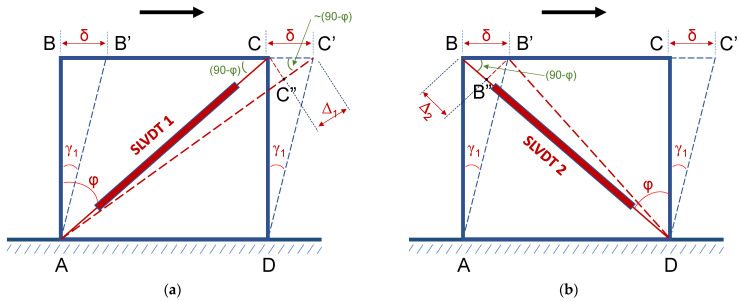
Elongation (**a**) and shortening (**b**) of the diagonals of the rectangular panel of the BCJ. Two diagonal SLVDTs were mounted on each specimen’s joint area to measure the elongation and the shortening of its diagonals at each loading step.

In this study, shear deformations developed to the joint during cyclic testing of the BCJs were measured using two SLVDTs that were diagonally mounted on the joint panel. Thus, the SLVDTs could record the elongation (Figure 4a) and the shortening (Figure 4b) of the diagonals of the joint panel. Shear deformations were calculated based on the diagonal elongation (Δ_1_ in Figure 4a) and the diagonal shortening (Δ_2_ in Figure 4b) of the joint rectangular panel using the SLVDTs’ measurements. From the elongation Δ_1_ of diagonal AC, shown in Figure 4a, the shear deformation could be estimated as *γ*_1_ *= δ/h*_1_; where *h*_1_ *= L*_1_*cosφ*_1_ and *L*_1_ is the length of the diagonal SLVDT1. Further, from the triangle CC’C’’, it was deduced that:

*δ* = Δ_1_/cos(90 − *φ*_1_) = Δ_1_/sin*φ*_1_ (1)

and

*γ*_1_ = (Δ_1_/sin*φ*_1_)/(*L*_1_cos*φ*_1_) = Δ_1_/(*L*_1_ sin*φ*_1_ cos*φ*_1_) = 2Δ_1_/(*L*_1_ sin2*φ*_1_) (2)

In the same way, from the shortening, *Δ_2_*, of the diagonal BD and the triangle, BB’B’’, the shear deformation *γ_2_* was:

*γ*_2_ = 2Δ_2_/(*L*_2_ sin2*φ*_2_) (3)

Since the joint panel had a rectangular form, it could be considered that *L*_1_ and *L*_2_ were more or less equal (*L*_1_ *≈ L*_2_) and *φ*_1_ *≈ φ*_2_. 

Thereupon, letting *L = L*_1_ *= L*_2_ and *φ = φ*_1_ *= φ*_2_, the average shear deformation *γ_avg_* was approximated as:

*γ*_avg_*=* (*γ*_1_ + *γ*_2_)/2→ *γ*_avg_
*= (*Δ_1_ + Δ_2_)*/*(*L*sin2*φ*) (4)

**Figure 5:**
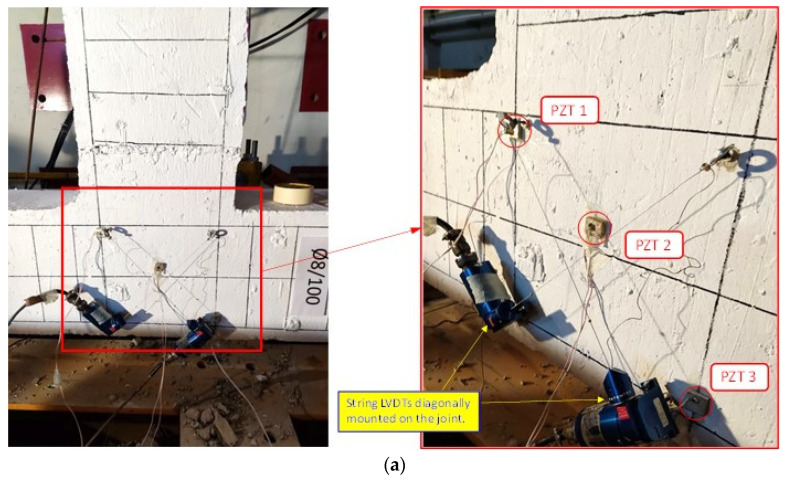

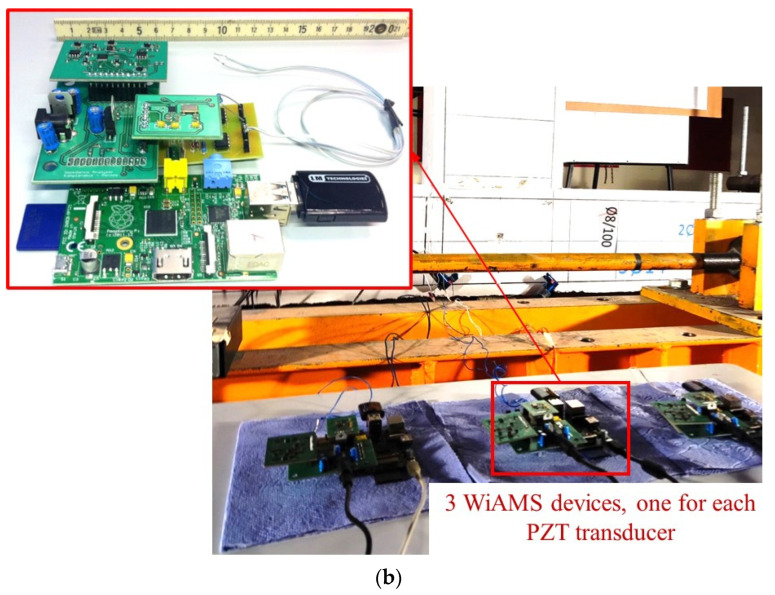
Measurement devices attached to specimen JC0: (**a**) Two diagonal SLVDTs and three epoxy-bonded PZT patches; (**b**) “Wireless impedance/Admittance Monitoring System (WiAMS)” devices, each one connected with two cables to the poles of each PZT patch mounted to the specimen, and detailed illustration of the small-sized SHM device.

**Figure 6:**
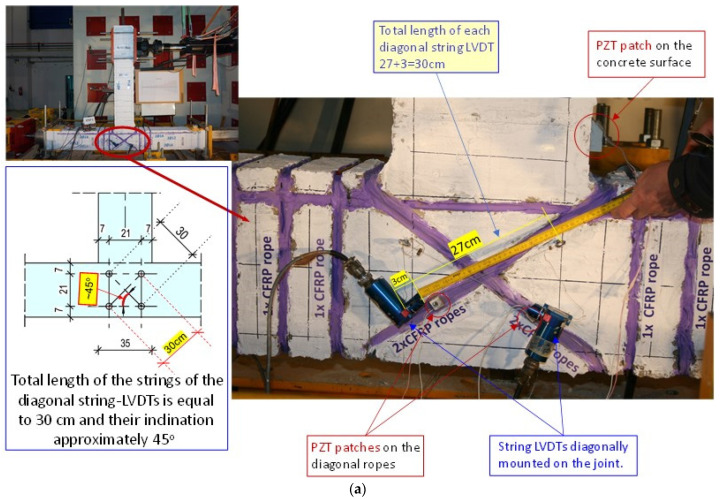

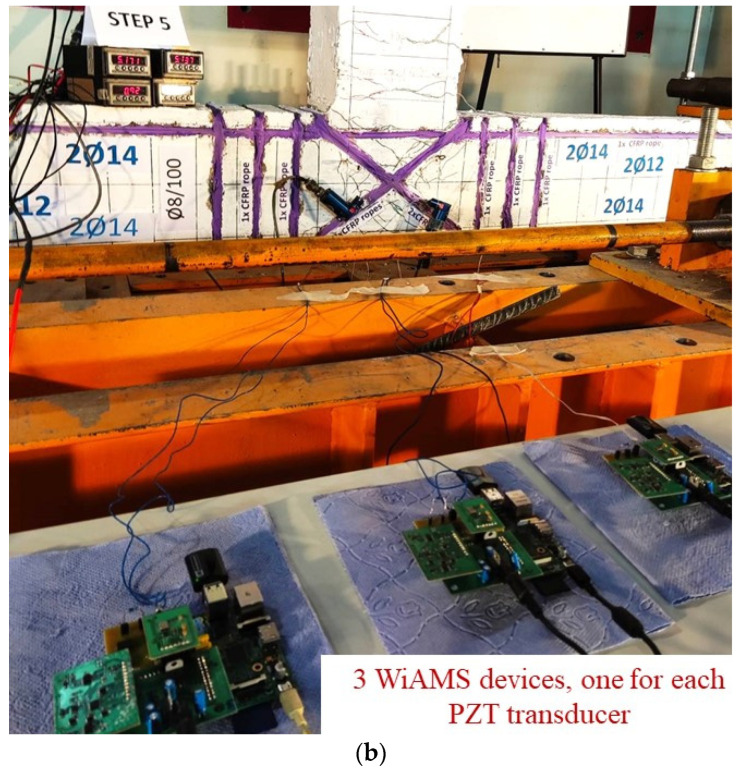
Measurement devices attached to specimen JB0F: (**a**) Three PZTs were epoxy-bonded; two on the diagonal C-FRP ropes and one on the concrete surface. Furthermore, two diagonal SLVDTs were mounted on the panel of the joint to measure the shear deformation at each loading step; (**b**) “Wireless impedance/Admittance Monitoring System (WiAMS)” devices, each one connected with two cables to the poles of each PZT patch mounted to the strengthened specimen.

The diagonal SLVDTs mounted on specimen JC0 and on specimen JB0F are shown in Figure 5a and Figure 6a, respectively.

## 5. PZT Transducers

During the imposed cyclic loading sequence of the tested BCJ specimens, damage due to concrete cracking, yielding of steel reinforcement and failures of the epoxy-bonded C-FRP ropes occurred. The severity levels of the damage were identified using the frequency responses of the mounted PZT transducers and measured by the used SHM device [54,55,56,57,58].

PZT plate sensors, with material mark designation PIC 151, dimensions 20 mm × 20 mm and a thickness of 2 mm, were used. According to the manufacturer’s specifications, it was a modified lead zirconate–lead titanate material with high permittivity, high coupling factor and high piezoelectric charge constant, suitable for low-power ultrasonic and low-frequency sound transducers. These sensors have a sputtered CuNi electrode and a negative electrode wrapped around the corner.

Before testing, three PZTs were bonded using epoxy adhesive on the concrete surface of specimen JC0 in the diagonal direction of the joint panel, as shown in Figure 5a. Three PZTs were also used in specimen JB0F; two PZTs were epoxy-bonded on the diagonal C-FRP ropes, and one on the concrete surface of the beam, as shown in Figure 6a.

The PZT-based SHM system used in this study is called the “Wireless impedance/Admittance Monitoring System (WiAMS)”, developed by Providakis et al. [55] and verified by various test projects in small- and full-scaled RC specimens, by the authors [56,57,58]. Two cables connected each WiAMS device to the poles of each PZT patch mounted to the RC BCJ specimens JC0 and JB0F, as shown in Figure 5b and Figure 6b, respectively.

The epoxy-bonded PZTs are first excited by a predefined sinusoidal frequency response generated and amplified by the signal generator module of the WiAMS devices, to be activated as actuators. The peak detector module of the WiAMS devices detects the peak voltage response value of the PZTs, which now act as sensors. Their output voltage frequency response is received by the WiAMS devices through their peak detector modulus and recorded as a peak voltage value. Data recorded by the PZTs are presented in voltage versus frequency diagrams. More details about the modules and the operation of the WiAMS device can be found in [54,55,56,57,58].

The PZT-enabled electromechanical impedance SHM techniques generate a surface electric charge in response to applied mechanical stress and undergo mechanical deformation in response to an applied electric field. Thus, when mounted on a structure the PZT patch actuator is actuated, and potential damage induces a change in the mechanical impedance (or its reverse admittance) of the structure, which reflects on the electrical signal of the PZT patch sensor. This way, recording the signal to the exciting frequency of a PZT transducer, the changes in its signal become an indicator of the presence of structural damage [59,60].

The interaction between the PZT patch and the structural integrity of a monitoring RC member is captured using the developed SHM that utilizes the WiAMS devices. Each device sends the interrogating waves through a PZT transducer and receives the reflected waves, simultaneously. This instrumentation offers remote control, high processing power, wireless data upload to an SQL database, email notifications, and iterative impedance magnitude estimations within a frequency range from 5 to 300 kHz with 1 Hz resolution.

Selection of the excitation frequencies of the mounted PZT patches is an important parameter affecting the effectiveness of the method and, therefore, special attention must be given to this. It has been proven that damage detection capability greatly depends on the successful frequency selection of the excitation, rather than on the voltage of the excitation loading itself. This observation demonstrates that excitation loading sequence can have a voltage level low enough that the technique may be considered easily applicable and effective for real structures. Thus, in this study, analyses were performed for a frequency range of 10 kHz to 250 kHz per step of 10 kHz by using one cycle per 10 kHz. This frequency range was chosen, based on the previous experience of the authors and researchers who investigated the suitable frequency range for concrete structures and members [43,61,62,63,64]. However, it should be noted that the plots presented herein and the index values were computed for signatures across the frequency range of 10–70 kHz (a subrange of 10–250 kHz in which signatures were recorded) because more cogent results have been traced from the signatures of this subrange.

The operation of the developed SHM system used the voltage output of the frequency generator and a resistor connected in series with the PZT patch of a circuit, and offered an efficient and straightforward method to measure impedance magnitude by exciting the device with a sinusoidal signal and measuring the amplitude of the voltage. Further, the peak voltage signal of the PZT transducer was directly dependent on the impedance magnitude of the PZT at radial frequency [61,62]. Thus, any change in the integrity state of the host RC members, due to structural damage, caused a corresponding shift in the peak amplitude of the voltage signal across the PZT transducer. This procedure resulted in modifications of the recorded PZT voltage frequency response at every increasing loading/damage level, compared to the response recorded at the end of the previous loading step. Thereupon, the magnitude of the observed frequency response changes might characterize the level of internal or external damage that occurred in the material during the loading step or until the loading step, respectively.

It was noted that a PZT was stimulated with a pulse within a frequency range and therein was a subrange where resonance took place. Resonance occurs when a system is able to store and easily transfer energy and, obviously, at this point even small differences in oscillation become more easily traceable. Thereupon, the differences between the lines, as recorded by the PZTs at the end of each loading step and at the beginning of the loading, were examined within the resonance frequency range.

## 6. Results

### 6.1. Hysteretic Responses and Energy Dissipation

**Figure 7:**
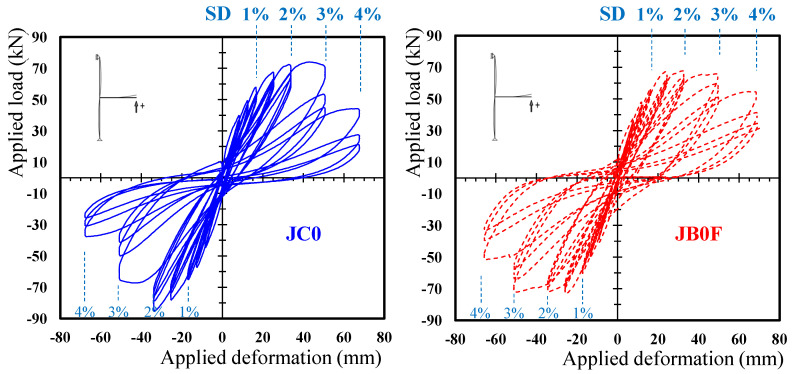
Hysteretic performance of subassemblages JC0 and JB0F.

**Figure 8:**
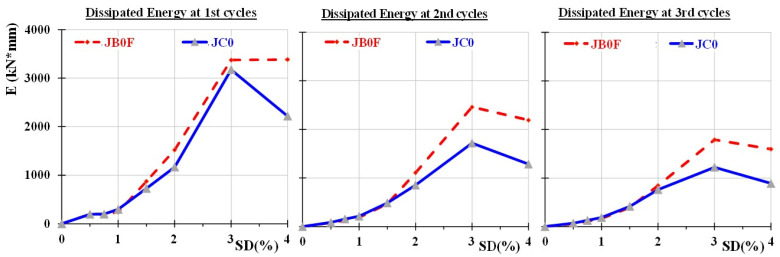
Comparative presentation of the dissipated energy of the two specimens in terms of the area of the hysteretic cycles. It was observed that the dissipated energy values of the strengthened specimen JB0F with the C-FRP ropes were, in all cycles, higher than the corresponding ones of specimen JC0 without C-FRP ropes.

The hysteretic performance of the two tested joint subassemblages are presented in Figure 7 in terms of applied load versus SD curves. Further, the dissipated energy values per loading cycle of the tested joint specimens, in terms of the area of the hysteretic cycles, are presented in Figure 8 for the 1st, 2nd and 3rd cycles of the imposed loading steps. From this figure, it can be observed that the dissipated energy values of the specimen JB0F with the C-FRP ropes were, in all cycles, higher than the dissipated energy of specimen JC0.

From the hysteretic response of specimen JC0, presented in Figure 7, it was observed that the maximum loads of hysteretic cycles at the 4th and 5th loading steps were substantially lower than the maximum loads of the previous step (3rd step). Thereupon, it might be concluded that major damage took place at these steps.

**Figure 9:**
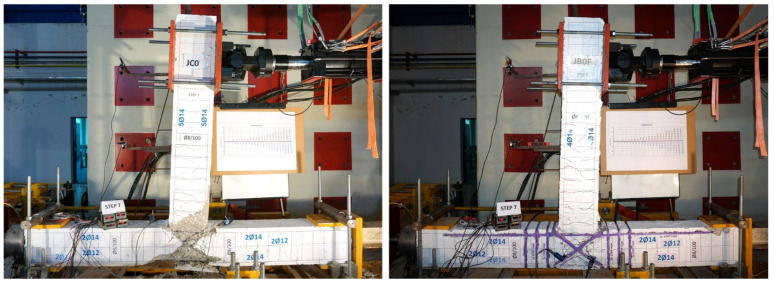
Comparative presentation of the damage of the two specimens after the test. In the case of the specimen JB0F the strengthening of the column and the joint body with C-FRP ropes kept the joint almost intact reducing the cracking.

Further, in Figure 9 the damage level severity of subassemblage JC0 is presented and compared to the corresponding damage level of the specimen JB0F, both at the end of the testing procedure (end of loading step 7). From the cracking performance of the specimens illustrated in this figure, severe diagonal cracks and total joint failure occurred in the joint body of specimen JC0, whereas cracking and concrete damage in the joint body of specimen JB0F was apparently lower. Therefore, it was obvious that the applied C-FRP ropes had efficiently improved the seismic capacity of the subassemblage and kept the joint body more or less intact.

### 6.2. Joint Shear Deformations as Measured by the Diagonal SLVTDs

**Figure 10:**
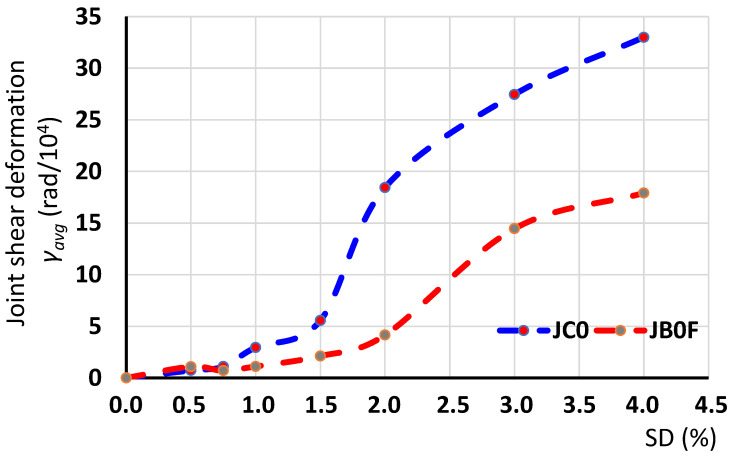
Shear deformations of the joint of the tested subassemblages JC0 and JB0F. Specimen JC0 exhibited higher values of shear deformation than specimen JB0F. This was more conspicuous at higher SDs (>2%) and it was attributed to the favorable influence of the diagonally applied C-FRP ropes.

The maximum absolute values of the shear deformations of the joint panel versus the SD of the examined BCJ subassemblages, as measured using the diagonal SLVDTs, and calculated, based on the relationships (1)–(4), are presented in Figure 10. In this figure, the maximum absolute average values of the joint shear deformation γ_avg_ (in rad) of specimen JC0, as calculated at each loading step of the test procedure, were compared to the corresponding ones of the strengthened specimen JB0F.

As can be observed from the diagrams of Figure 10, specimen JC0 exhibited higher values of shear deformation than specimen JB0F. This was apparently attributed to the favorable influence of the diagonally applied C-FRP ropes in the joint of specimen JB0F. This became more conspicuous at high SDs (>2%), since at these loading steps the mounded C-FRP ropes kept the joint body intact and efficiently reduced the shear deformation. From the joint shear deformation versus SD diagrams of specimen JC0, presented in Figure 10, it is also apparent that the highest values of shear deformations were attained in the 4th and 5th loading steps, with SD = 1.5% and 2%, respectively. Considering that there were large differences with the shear deformation of the previous steps it was concluded that significant damage took place at these steps.

### 6.3. Steel Bar Strain as Measured by Strain Gauges

**Figure 11:**
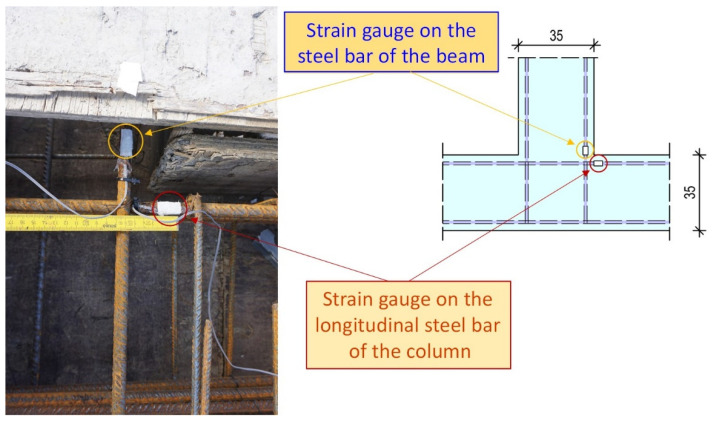
Strain gauges were bonded on the steel reinforcing bars of the beam and the column at the edge of the joint body panel of each specimen.

**Figure 12:**
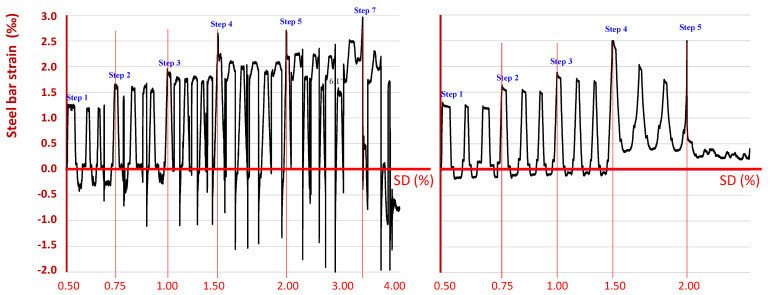
Comparative presentation of the strain history of a column longitudinal steel bar during the testing procedure of the two specimens (JC0 and JB0F), as recorded by strain gauges epoxy-bonded on the steel bars of each specimen.

Strain gauges were mounted on the steel longitudinal reinforcing bars of the beams and the column near the joint body of each specimen, as shown in Figure 11. Comparing the strain values, as recorded by the strain gauge on the steel bar of specimen JC0, with the strain values as recorded by the strain gauge on the corresponding longitudinal bar of specimen JB0F, it was observed that they were substantially higher, especially in the negative direction (Figure 12). Further, it was also noted that the steel bar in specimen JC0 developed both tensional and compressional strains. This eventually caused a dense crack system in the joint body (see also the cracking patterns at the failure of specimen JC0, in Figure 9).

On the contrary, in the case of specimen JB0F, lower values of strain were recorded during the testing procedure and, further, the strain did not appear to attain equally high values in both directions. This observation was in good compliance with the final state of specimen JB0F, considering the limited extent of the final cracking system and the low level of the whole damage, as can be seen in Figure 9.

### 6.4. Damage Diagnosis Based on the PZT Transducers

**Figure 13:**
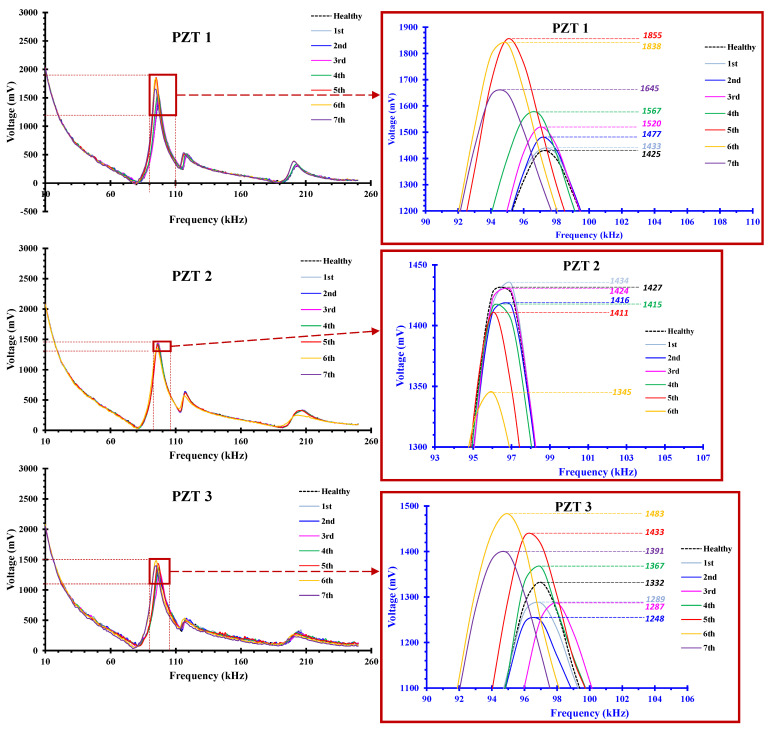
Frequency response of PZT transducers attached to the concrete surface in the direction of the joint panel diagonal of specimen JC0. Each curve represents the voltage versus frequency of the PZT at the end of each loading step (unloaded condition).

**Figure 14:**
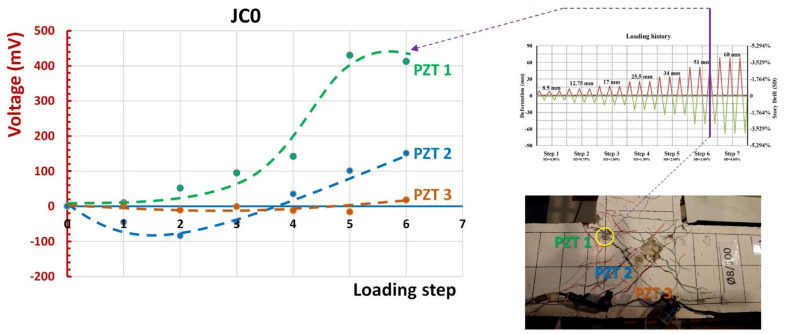
Voltage differences between the peak voltage value recorded at each loading step minus the voltage at the same frequency in the healthy condition, representing the level of the damage that occurred in the vicinity of the PZT patch. PZT 1 transducer at the 5th and 6th steps of the loading procedure exhibited substantial increase in the voltage output indicating significant change/damage, as can be verified in the photograph of the cracking pattern of specimen JC0 after the imposed loading sequence.

**Figure 15:**
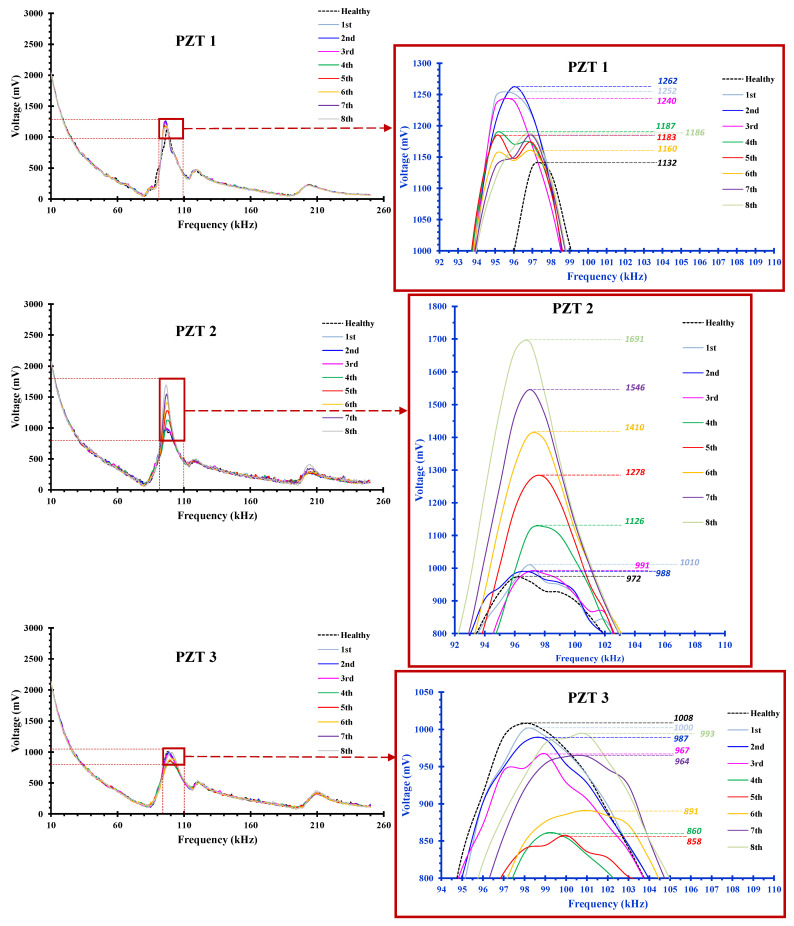
Frequency response of the PZT transducers attached to the concrete surface of the beam near the joint body (PZT 1) and to the surface of the C-FRP ropes that were diagonally epoxy-bonded on the panel of the joint body (PZT 2 and PZT 3) of the strengthened specimen JB0F. Each curve represents the voltage versus frequency of the PZT at the end of each loading step (unloaded condition).

**Figure 16:**
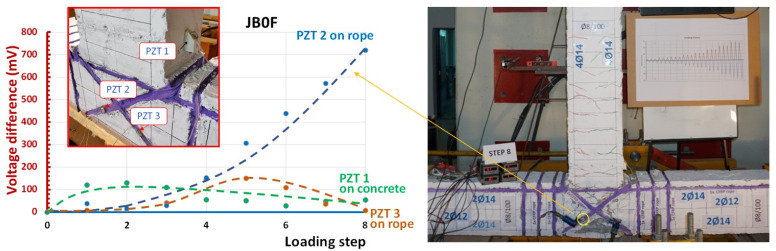
Voltage differences between the peak voltage value recorded at each loading step minus the voltage at the same frequency in the healthy condition represent the level of the damage that occurred in the vicinity of the PZT patch. The PZT 2 transducer attached to the C-FRP rope after the 5th loading step exhibited substantial increase in the voltage output indicating significant change/damage, as can be verified in the photograph of the cracking pattern of specimen JB0F after the imposed loading sequence. Concrete in the beam area near the joint remained more or less intact confirming the neglected voltage difference derived from the output of the PZT 1 transducer, which was epoxy-bonded on beam concrete surface.

The results of damage diagnosis, based on the PZT patches epoxy-bonded on the BCJ subassemblages, are presented in Figure 13, Figure 14, Figure 15 and Figure 16. Specifically, Figure 13 and Figure 14 concern specimen JC0 and Figure 15 and Figure 16 concern the strengthened one, JB0F.

In specimen JC0, three PZTs were bonded on the concrete surface in the direction of the diagonal line of the joint body panel (see also Figure 5a). Signals recorded by these PZTs are presented in Figure 13, in terms of voltage versus frequency response curves. Each curve of the diagrams represents the output data of the PZT at the end of each loading step at the unloading condition.

Each loading step brought about changes in the structure resulting in changes in the position and the shape of the recorded curve, compared to the one recorded at the beginning of the loading procedure (healthy condition). Therefore, it might be assumed that the frequency response magnitude changed at the end of each loading step, compared to the baseline one which corresponded to the structure without any damage. This way, the discrepancies observed in the frequency response curves at each loading step represented the level of internal or external damage that occurred in the joint area until that loading step, reflecting the damage state of the material in the vicinity of the PZT.

The peak voltage values of the PZT transducers at the end of each loading step, obtained in the frequency range 90–100 kHz, were reported, and are highlighted in the zoom-in diagrams of Figure 13. The differences of the peak voltage values at each loading step minus the corresponding voltage at the same frequency at the beginning of the loading procedure (healthy state) was calculated, and are presented in Figure 14.

PZT 1 transducer at the 5th and 6th steps of the loading procedure showed a substantial increase in the voltage output, indicating severe change in the state of the material. It was diagnosed that, at this stage, significant damage occurred in the neighborhood of PZT 1, as can be seen in the photograph of Figure 14. Nevertheless, peak voltage differences of PZT 3 failed to indicate damage accurately, since their values were very low, although substantial damage in concrete occurred due to spalling during the highest loading steps. In this case, the implementation of a proper statistical index was required to quantify the damage level, considering the discrepancies between the entire frequency response curve of the healthy state and the curve of examined loading steps [43,56].

Concerning the retrofitted BCJ specimen JB0F, three PZT transducers were implemented; one PZT patch was epoxy-bonded on the concrete surface of the beam and two PZTs were also epoxy-bonded on the diagonal C-FRP ropes applied in the joint panel (see also Figure 6a). Figure 15 illustrates the output signals of these PZTs, in terms of voltage versus frequency response diagrams, and each curve represents the recorder data of the PZT at the end of each loading step at the unloading condition.

Regarding the frequency response of the PZT 1 transducer (see also Figure 15) relatively slight discrepancies could be detected between the curves representing the examined loading steps and the baseline curve (healthy state) of the structural member. Similar decays between the frequency response curves of the used PZT were also produced in relative experimental studies conducted by the authors in [56,57,61,62] and other researchers in [65,66,67]. This fact indicates that there were no significant changes in the material state in the neighborhood of the attached transducer, and, therefore, no considerable damage or cracking was detected in the concrete of the beam near its conjunction with the joint by the SHM measurement of PZT 1.

Helpful information can also be derived from Figure 15 and the frequency response data recorded by transducers PZT 2 and PZT 3 attached to the C-FRP ropes that were epoxy-bonded diagonally on the joint panel. First, the PZT 2 transducer exhibited substantial discrepancies concerning the peak values and the overall shape of the frequency curves measured at the end of each loading step, compared to the baseline curve of the healthy state recorded at the beginning of the loading procedure. Further, the zoom-in diagrams of Figure 15 focused on the frequency range 90–105 kHz reveal the higher differences in the peak voltage values at each loading step measured from the PZT 2 transducer than the corresponding differences derived from the measurements of the PZT 1 transducer. It was also noted that the greater values of these differences were obtained from the PZT 2 transducer during the 4th loading step with SD = 1.5% and, especially, after the 5th loading step with SD = 2%. This fact revealed potential excessive damage to the C-FRP rope due to fiber debonding or micro-fracture that occurred right after this SD value.

Furthermore, although the PZT 3 transducer was symmetrically placed to PZT 2 and attached to the diagonal C-FRP rope, the frequency response of PZT 3 seemed quite different from that of PZT 2. The diagrams of PZT 3, shown in Figure 15, demonstrate relatively slight discrepancies between the frequency responses recorded at the end of each loading step and the baseline curve. Nevertheless, this variation could be justified, up to a point, by the measurements of the column longitudinal steel bar strains during the cyclic testing procedure, shown in Figure 12. These strains were not symmetrical in both directions, which revealed that the loading cycle with positive (+) direction (see also Figure 3b for notation) caused initial tension and more critical damage to the C-FRP rope with the PZT 2 transducer than the C-FRP rope with PZT 3 (see also photographs of Figure 16 for notation).

Figure 16 presents the diagrams of the voltage differences per loading step of all three transducers of specimen JB0F calculated from peak voltage values at each loading step minus the corresponding voltage at the same frequency at the beginning of the loading procedure (healthy state). These diagrams, and the photograph in Figure 16, that illustrates the cracking pattern of the tested BCJ subassemblage after the imposed loading sequence, verify the previous remarks.

The comparisons between the observations derived from the hysteretic and the cracking performance of the tested BCJ specimens and the results of the proposed SHM technique also revealed a helpful conclusion. Damage severity of the BCJ specimens could be identified by the applied load versus deformation curves and the crack propagation of the structural member during the imposed cyclic loading. However, applying the proposed PZT-based SHM technique offered real-time surveillance, continuous inspection and in-situ measurement for far-field damage identification and early warning of imminent failure in real-life RC frame buildings.

The measurements acquired using the conventional instrumentation by SLVDTs and strain gauges and the corresponding test data derived from the PZT transducers and the proposed SHM procedure provided strong indications of damage diagnosis throughout the entire imposed cyclic loading sequence. Nevertheless, the results of the proposed PZT-based SHM technique offer two more advantages: (a) reliable continuous non-destructive inspection and damage assessment from a distance and (b) prompt and real-time evaluation of damage degree with prompt warnings of early indications of critical failures at initial damage stages, such as the onset of diagonal shear cracking in existing real-life in-service RC infrastructures. This allows the supervising engineer to interrupt building operation services to prevent further deterioration and decrease the risk of catastrophic failures.

## 7. Summary and Conclusions

(a) The application of PZT transducers to study the efficiency of an innovative strengthening technique for RC columns and BCJs was presented and discussed. Two real-scale RC BCJs were tested in lateral cyclic loading. The second specimen was strengthened with C-FRP ropes externally applied and epoxy-bonded on the column and the joint body.
(b) Successful damage diagnosis was achieved, based on data from PZT transducers epoxy-bonded on the concrete surface and C-FRP ropes. The results were verified using measurements of the shear deformations of the joint body from diagonally placed SLVDTs. Furthermore, the strains of the steel bars, as measured with strain gauges, also confirmed the damage diagnosis attained by the frequency responses of the PZTs.
(c) Frequency response data acquired by PZT transducers, SLVDTs and strain gauges established the effectiveness of the applied retrofitting technique using diagonal C-FRP ropes externally epoxy-bonded on the column and, especially, to the joint panel of the tested BCJ specimens.
(d) The proposed PZT-based SHM system seemed efficient in identifying the location and the severity level of damage using the voltage differences of the peak voltage values at each loading step minus the corresponding voltage at the same frequency at the beginning of the loading procedure (healthy state). The influence of distance, material (concrete or C-FRP rope) and damage level triggered changes in the output signals of the PZT transducers, seeming to be a reliable assessment tool for damage quantification.
(e) The application of a network of PZT transducers with the implementation of advanced, portable and wireless SHM devices could offer real-time surveillance, continuous inspection and in-situ measurement for far-field damage identification and early warning of imminent failure in real-life critical structural members of RC frame buildings. Based on research already conducted, promising results, concerning the ability of the developed SHM system to provide early indications of failure at initial damage stages, were derived.
(f) Reported results concerning the effectiveness of the developed PZT-based SHM system have so far been generated in laboratories. However, field monitoring of existing in-service RC infrastructures is required. Thus, future research to bring the developed damage detection method from laboratories to real-life applications is advocated.
(g) Installation procedure and positions of the PZT transducers mounted to the critical regions of the monitored structural members significantly influence the capability and accuracy of the SHM technique. The extent and the precision of the performed damage diagnosis are also parameters that should be thoroughly investigated in future works.
(h) The conducted research concentrated on concrete damage detection. However, for future research, more attention should be paid to steel or composite reinforcement damage identification, such as yielding, bond-slip or debonding failures, to issue early warnings before a fatal collapse happens.
(i) Proper quantification of damage assessment is required using statistically scalar damage indices for evaluating the damage severity in the examined RC structural member. Performing advanced artificial neural networks as quantitative approaches is also an important research topic.

## Data Availability

The data presented in this study are available on request from the corresponding author.
